# Changes in HIV knowledge, and socio-cultural and sexual attitudes in South India from 2003-2009

**DOI:** 10.1186/1471-2458-11-S6-S12

**Published:** 2011-12-29

**Authors:** Janet Bradley, S Rajaram, Stephen Moses, Parinita Bhattacharjee, Anil M Lobo, BM Ramesh, Reynold Washington, Michel Alary

**Affiliations:** 1 CHARME-India Project, Bangalore, India KHPT office, IT Park 5th floor, #1-4 Rajajinagar Industrial Area, Behind KSSIDC Admin Office, Rajajinagar, Bangalore 560 044, India; 2 Centre hospitalier affilié universitaire de Québec, Unité de recherche en santé des populations, Centre de recherche du CHA de Québec, Hôpital du Saint-Sacrement, 1050 chemin Ste-Foy, Québec G1S 4L8, Canada; 3 Karnataka Health Promotion Trust, Bangalore, India, IT Park 5th floor, #1-4 Rajajinagar Industrial Area, Behind KSSIDC Admin Office, Rajajinagar, Bangalore 560 044, India; 4 Centre for Global Public Health, Faculty of Medicine, University of Manitoba, 771 Mc Dermot Avenue, Medical Rehabilitation Building, Room R070, Winnipeg, Manitoba R3E 0T6, Canada

## Abstract

**Background:**

As communities face serious pressures on traditional values, such as those posed by HIV infection, cultural inertia may result, whereby existing trends towards more liberalized views of sexuality are stalled. We examined changes in attitudes around HIV in Bagalkot district, south India, between 2003 and 2009.

**Methods:**

General population surveys were conducted in 2003 and 2009, among approximately 6,600 randomly sampled men and women in 10 villages and 20 urban blocks of Bagalkot. Questions about HIV knowledge, sexuality, gender and condoms were included. We compared responses in the two surveys using a multivariate logistic regression model.

**Results:**

Awareness of HIV increased significantly from 76.9% in 2003 to 87.8% in 2009, and condom awareness increased significantly (37.4% to 65.4%) in all groups studied. However, in 2009, only 23% of people mentioned condoms as a means of prevention, an improvement from 8% in 2003 (adjusted odds ratio (AOR) 3.3; 95%CI 2.6-4.1, p <0.001). There was a significant increase in the number of women believing sex workers should be compulsorily tested for HIV (76.3%-86.4%%, AOR 1.8; 95%CI 1.4-2.4, p<0.001). An increasing number agreed that “it is wrong to talk about sex” (p=0.05), especially women (21.9% vs. 32.4%, p<0.01). There was an increase in women who thought it “wrong to talk about AIDS in a respectable family”, and more respondents in 2009 thought it improper to discuss condoms (15.6% vs. 27.4%, AOR 1.9, 95%CI 1.4-2.8, p=0.001). In 2003, 31.4% agreed that “access to condoms promotes promiscuity”, increasing to 45.2% in 2009 (AOR 1.7; 95%CI 1.3-2.3, p<0.001). Educated and young urban women were the most likely to believe this. In 2003, 19.3% and in 2009 30.2% (AOR 1.8, 95%CI 1.4-2.3, p<0.001) thought that sex education promotes sexual activity and promiscuity.

**Conclusions:**

Despite increases over time in HIV-related knowledge and reductions in stigmatizing attitudes, resistance to changing cultural mores was apparent, with less willingness to embrace openness and discuss sexuality. Young and female respondents appeared to be the most resistant to change, reflecting a cultural inertia that mirrors studies of other pressures on traditional societal values. More effort is required to advocate among women and young people for healthy sexuality, openness and safe sex practices.

## Background

Although HIV/AIDS in India has been present for more than two decades, news of the epidemic has been slow to reach some communities; in the third National Family Health Survey in 2005-6 (NFHS-3), 30% of women in Karnataka said they had never heard anything about HIV/AIDS [1]. Life in rural communities of northern Karnataka in particular has been changing recently as there are increasing educational opportunities and non-traditional employment, increasing access to television and sexually explicit movies, increasing female autonomy and freedom from frequent childbearing (2005/6 total fertility rate 2.1) [1]. There is also widespread use of contraception (NFHS-3 contraceptive prevalence rate 64%, up from 58% in NFHS-2) [1], a feature that generally is associated with sexual emancipation [2]. Despite these changes, this (mostly Hindu, partly Moslem) society is still conservative and traditional in northern districts of Karnataka. The age at marriage is still quite low for women with 42% of women aged 20-24 in 2005/6 married before the age of 18 [1]; and 42% of ever-married women aged 15-49 years in 2007/8 were illiterate [3]. Regressive attitudes to women prevail: two-thirds of women and men in Karnataka in 2005 thought that it was justifiable for a man to beat his wife in some circumstances [1]. Sex education and contraception information for schoolchildren was acceptable to less than half of women interviewed nationally in the NFHS-3, although almost everyone agreed that children should be taught “moral values” in school [4].

Against this conservative socio-cultural-sexual backdrop, Karnataka ranks third in India among the states most affected by HIV/AIDS. In 2005/6, an estimated 0.28% of adults aged 15-49 nationally and 0.69% of adults in Karnataka were infected with HIV [4]. Sentinel surveillance at antenatal clinics in 2006 identified 15 districts in the country with a prevalence of more than 3.0%, three of which were in Karnataka [5]. A study in districts of northern Karnataka in 2001/2 also revealed considerable heterogeneity in HIV risk *within* districts, associated with clustering of mostly traditional caste-based sex workers in certain locations [6]. In 2003, a general population survey in three talukas (sub-districts) in Bagalkot district in northern Karnataka, found an overall HIV rate of 3.2% . In 2009, a repeat general population survey in Bagalkot found that HIV prevalence overall had fallen to 2.6%, with the greatest (and statistically significant) decline observed in the 15-24 year age group [7]. This may have been due in part to intensive targeted intervention programmes in the area over the past 10 years, focused on female sex workers and clients [8,9]. These two general population surveys have also provided data on community members’ knowledge and attitudes, which are examined in this paper.

Sociological literature on changes in societies faced with new pressures on social order such as an HIV epidemic point to several features. First, there is an instinctive fear of the unknown or fear of contagion, which may be related to poor knowledge on how to prevent HIV [10-13]. Second, societies tend to attempt to blame the victims by identification of “folk devils” or scapegoats [10,13-18] with moral and sexual panic whipped up also by media [13-16,19,20]. There may also be an attempt to explain the collapse of morality with religious ideas that focus on God’s wrath, especially in deeply religious Hindu/Moslem societies as this one [20,21]. The enactment of this fear and blame leads to stigma and discrimination, and “blaming the victim” especially with respect to already stigmatized or sexually progressive groups (sex workers, men who have sex with men, women asserting financial and sexual independence, etc.) [10,12,13,16,22-27]. A feeling is often manifested that the health threat emanates from more liberal sexuality and amoral behaviour, and that the very fabric of society is being threatened by the “slippery slope” of moral decay [2,15,16,20,28]. This perception often results in increasingly conservative attitudes, a return to “cultural” or traditional moral values, and interestingly is often found more in youth, women and the educated [2,19,20,28]. Paradoxically, there may be decreased acceptance of the very modalities that might help reduce the panic (e.g. sex education, discussion of condoms) [16,17,20].

In this paper, we examine changes in responses to knowledge and attitudes around sexuality and HIV among members of the general population in surveys conducted in Bagalkot district in northern Karnataka in 2003 and 2009, using a framework shown in Additional file 1 that reflects this literature.

## Methods

In 2003 under the Indian-Canada HIV/AIDS Project (ICHAP), the Population Research Centre, Dharwad, Karnataka, undertook a cross-sectional general population survey to study HIV prevalence and sexual behaviours in 10 villages and 20 urban blocks (neighbourhoods) in three *talukas* (sub-district administrative units) of Bagalkot district, selected purposively as they were thought to be *talukas* with high HIV prevalence where interventions were later planned. Detailed methods used in this survey are available elsewhere [29]. Briefly, India’s 1991 census list of villages was used as the sampling frame for randomly selecting 10 out of 237 villages based on the probability proportional to population size (PPS) method of sampling [30]. The National Sample Survey Organization’s (NSSO’s) sampling frame of urban census enumeration blocks for the period 1997-2002 was used for selecting the required number of urban areas. The selection of urban blocks was based on a systematic random sampling procedure. The 2009 study was conducted in the same rural and urban areas selected in the 2003 study, with the exception of six urban blocks in Bagalkot town that had to be replaced as they had been submerged under a newly constructed reservoir; six replacement blocks were selected using the NSSO sampling frame for the period 2002-7. The target sample size in both 2003 and 2009 was 3300 in urban areas and 3300 in rural areas, with approximately equal males/females aged 15-49. The total sample size was derived based on factors such as the expected level of non-response, cost and time of conducting such studies and the desired level of precision in estimating the prevalence of HIV and sexually transmitted infections in the general population at the district level.

In each study period, a complete household census was first undertaken in all 30 sites to provide a basis for random selection of respondents aged 15-49, for face-to-face interviews. The actual respondents were not the same respondents as in 2003, although there may have been some of the same people surveyed by chance. In each village the number of individuals selected was based on the size of the 15-49 year old population. However, in urban areas the number of individuals selected was fixed at 165 per urban area. Interviews were conducted by trained staff and questions were asked about HIV and safe sex practices, attitudes to gender and sexuality, attitudes to condoms, feelings about the causes of HIV, attitudes towards people with HIV/AIDS, and attitudes towards increased access to condoms and sex education.

Sample weights were calculated based on design weights, adjusted for the effect of differential non-response in each primary sampling unit. Data analysis was undertaken using STATA version 10 (Stata Corporation, USA). The differences between 2003 and 2009 were ascertained using multivariate logistic regression controlling for age, sex, education, religion, marital status, urban/rural location and taluka, where appropriate, with adjusted odds ratios (AORs), 95% confidence intervals (95%CIs) and p-values for each subgroup calculated.

## Results

### Sample structure

The major difference between the two surveys is that at the time of the first survey there were few HIV prevention activities in the area and there was a certain amount of mistrust in the community to interviewers; by 2009, the situation had changed dramatically; HIV was no longer a new concept for the communities and people responded more favourably to the survey. The response rate therefore increased from 74% in Round 1 to 82% in Round 2. In total 4,949 people were interviewed in 2003 and 5,383 in 2009. There were no significant differences in the social and demographic characteristics of the respondents in the two surveys, except that in Round 2 respondents had higher educational attainment [7].

### HIV and sexuality knowledge

By the time of the second survey in 2009, almost all respondents (87.8%) said they had heard something about HIV/AIDS, an increase from 76.9% in 2003, and this was observed in all age and education groups (see Additional file 2) and in rural and urban settings (see Additional file 3). The lowest level of awareness was in men with a low level of education (76.8%). The most significant increase in awareness over time was seen in urban women (AOR 3.5, 95%CI 1.6-7.2, p=0.002). Condom awareness also increased significantly, from 37.4% to 65.4%, and in all groups, although in 2009 only 48.6% of women reported that they had heard about or seen condoms. Rural women and uneducated women were the least likely to know about them (33.1% among women with less than 4 years of education), although this was a significant increase over the 6.6% in this group observed in 2003 (AOR 7.4; 95%CI 5.5-9.8, p<0.001). Condoms are promoted as a cornerstone of HIV prevention, and yet after several years of national and local campaigns only 23.0% of people mentioned them spontaneously as a means of preventing transmission, although this was a significant increase over the 8.3% observed in 2003 (AOR 3.3; 95%CI 2.6-4.1, p<0.001). Urban and well educated males, and men between the ages of 25-34 were the most likely to mention them, and young poorly educated and rural women were the least likely to do so. The proportion of individuals who spontaneously mentioned limiting the number of sexual partners as a prevention measure was actually significantly less in 2009 than in 2003 (31.5% vs. 23.3%, AOR 0.6; 95% CI 0.4-0.8, p=0.04). The reduction was mostly in males and among better educated men and women. About 20% of respondents (and only slightly less than those mentioning limiting partners) reported avoidance of injections as a means of prevention in both surveys. Respondents with the highest levels of education were the most likely to think that injections should be avoided to prevent HIV.

We hypothesized that modernity and more open discussions around sexuality might have changed community perceptions about non-harmful practices such as masturbation; in 2003 30.5% of respondents thought this practice harmful. However, fear that this practice is harmful actually increased to 38.1% of respondents (49.8% of men) in 2009, with both urban and rural men equally fearful of negative health effects. The most significant increase was observed for women in rural areas (p<0.001). The most educated men and young people were just as likely to misconstrue this as uneducated and older men.

### Attitudes towards HIV infected people

We examined six aspects of stigmatizing attitudes to people with HIV by reading statements that required respondents to agree or disagree (see Additional files 4 and 5). The first statement related to the idea that AIDS is a punishment from God for sins committed. In 2003, 43.5% of those who had heard of HIV believed this to be true, but this figure declined significantly to 28.9% in 2009 (AOR 0.5; 95% CI 0.5-0.6, p<0.001). Although the decline was significant in all groups, rural people persisted in this belief more than urban people, as did uneducated respondents and older women. More than half the respondents in all groups agreed that those who had sex outside marriage deserved to get AIDS, a figure that did not change overall from 2003 (61.6% vs. 60.7%). Older men and urban men were the most likely to have relaxed their views on this over time. Sex workers were also more likely to be identified as key actors in the epidemic and targets for mandatory interventions, especially among some groups; the greatest increase in this belief was among young women (76.1% vs.86.8%, AOR 2.0, 95%CI 1.4-2.7, p<0.001), younger people, and the most educated classes, especially the most highly educated women (87.2% vs. 91.8%, AOR 1.7, 95%CI 1.1-2.4, p =0.01). This kind of stigma reduced the most among men aged 35-49 (90.5% vs. 79.3%, AOR 0.4, 95%CI 0.2-0.8, p= 0.007).

Despite this identification of particular scapegoats, general stigmatizing attitudes to infected people appear to have declined. In 2003, almost one-half of respondents who had heard about HIV, felt that infected people should be thrown out of the community to prevent spread of the disease, and this fell significantly in all groups, to 27.9% overall in 2009 (AOR 0.5, 95% CI 0.4-0.6, p<0.001). Women were more likely than men, older people more likely than younger people, rural dwellers more than urbanites, and uneducated people more likely than educated people to hold these regressive views, with rural, older and uneducated women the least sympathetic, and urban men, men aged 25-34, and educated women the most progressive.

Fear of households with HIV infected people and a proxy for stigma was measured by asking if respondents agreed that one should *not* take a bride from a family that included infected people. There was a significant drop in the numbers agreeing with this statement, from 49.5% in 2003 to 32.1% in 2009 (AOR 0.5; 95%CI 0.4-0.6, p<0.001). This perception was held equally by all age groups, although rural males were the least progressive on this count and urban males the most progressive. The greatest reduction in this discriminatory and stigmatizing opinion was among the most educated groups. In 2003, 38.2% of people with more than grade 10 education agreed with the statement, compared to 24.9% in 2009, a significant decline (AOR 0.5; 95%CI 0.4-0.7, p<0.001). Although such stigma had reduced in all age groups, young people were just as likely as older people to hold this view.

Discrimination against children with HIV also fell significantly over time in all groups studied. The proportion of respondents feeling that HIV positive children should have separate schools fell significantly from 41.8% to 28.2% (see Additional files 4 and 5), with reductions in stigma among both males and females, and in both urban and rural areas. Educated people and young people (especially young females) showed much more tolerance for this position in 2009 than did older people; the proportion of women under 25 who felt that HIV positive children should have separate schools fell significantly between the two surveys (44.7% vs. 22.5%, OR 0.4; 95%CI 0.3-0.5, p<0.001).

### Attitudes around sexuality

Attitudes around female virginity are deeply ingrained in Hindu society with *kanya dan*, (the gift of a virgin) as a central tenet of marriage [31]. In our study we asked people if they thought women should be virgins at marriage and 87.4% agreed with this in 2009, although this was slightly less than the 90.1% in 2003 (Additional file 6). Women held this belief more than men; in fact among rural men the proportion believing this fell significantly from 87.3% in 2003 to 80.7% in 2009 (AOR 0.6; 95%CI 0.4-0.8, p=0.004), whereas slightly more rural women in 2009 than in 2003 thought this (see Additional file 6). As shown in Additional file 7, young males aged 15-24 and older males aged 35-49 were the most “liberal” about virginity in 2009 (81.0% and 80.4% respectively believing that women should be virgins at marriage), and were the most likely to have changed their views over time; and young women were the least likely to have changed their views (92.4% believing this notion); in fact, more agreed with the notion in 2009 than 2003. There were significant changes in attitudes to female enjoyment of sex. In 2003, almost one quarter of people thought that it was immoral for a women to seek enjoyment in sex, but this fell in from 2003 to 2009 (22.6% vs. 15.3%, AOR 0.6; 95% CI 0.5-0.9, p=0.01). Older people were the most likely to agree with this statement, although the views of women liberalized somewhat over time, whereas those of older men became more rigid. Rural women were the most conservative about female sexual enjoyment, although the proportion believing that women should not enjoy sex declined over time (34.1% vs. 19.1%). Urban men were the least conservative, as well as people under the age of 25 and the more educated respondents, although the greatest change over time was actually among the least educated women (37.5% vs. 19.8%, AOR 0.4; 95% CI 0.3-0.6, p<0.001).

### Attitudes towards openness in discussing sexuality and HIV

We asked three questions about issues such as sex, HIV and condoms that might be discussed between friends and family and that might lead to greater openness and understanding of the problems facing the community (see Additional files 6 and 7). First we read the general statement that “it is wrong to talk about sex”. Rather than dismissing this notion an increasing number of people agreed with this statement in 2009 compared to 2003 (24.2% vs. 29.2%, AOR 1.4; 95% CI 1.1-1.9, p=0.02). Although there was little change among men, women were much more likely to agree with the statement in 2009 than they had been in 2003 (21.9% vs. 32.4%, AOR 1.9; 95% CI 1.4-2.5, p<0.001), and this was true in both urban and rural areas. Rural women and women with low education levels were the ones most likely to agree with the statement; in 2009, 37.9% of women with less than 5 years of education agreed that it was wrong to talk about sex, compared with 22.7% in 2003, a significant increase (AOR 2.1; 95% CI 1.5-3.1, p<0.001). Young people had just as regressive views in this regard as older people.

We also asked participants if they thought it was wrong to talk about AIDS in a respectable family. Again, there was a significant increase in the proportion of people agreeing with this statement between the two rounds (21.4% vs. 25.2%, AOR 1.3; 95% CI 1.0-1.7, p=0.03). The most significant increase was seen in urban women, young people and more educated women; there was a significant increase in those agreeing with the statement among both males and females under the age of 25. In fact, young men and women became more conservative over time than older people with one quarter of respondents under the age of 25 agreeing in 2009 that it is wrong to talk about AIDS in a respectable family. The most educated respondents were more liberal than the least educated but were also the ones to have significantly changed their views regressively over time, especially the most educated women (12.2% vs. 23.8%, AOR 2.3; 95% CI 1.5-3.4, p<0.001). Young men too (aged 15-24), exhibited regressive tendencies over time with respect to openness about HIV discussions (18.4% vs. 28.2%, AOR 1.8; 95% CI 1.1-3.0, p=0.02). Reflecting a greater negativity towards openness over time, more people in 2009, of those who had heard of condoms, thought it not proper for respectable people to talk about them (15.6% vs. 27.4%, AOR 1.9; 95% CI 1.4-2.8, p=0.001). Rural people were more likely to think this than urbanites, as well as those with low education levels; young people found open discussion of condoms just as distasteful as older people in 2009, and in all age groups this increased over time.

It is generally believed that discussion of safe sex leads to safer sex rather than to promiscuity. When we asked our Bagalkot respondents to comment on two statements in this regard (“easy access to condoms promotes promiscuity” and “sex education increases sexual activity and promiscuity”), there was a significant increase in the number of people agreeing with this. In 2003, 31.4% overall agreed that access to condoms promotes promiscuity, and this increased significantly to 45.2% in 2009 (AOR 1.7; 95% CI 1.3-2.3, p<0.001). In all groups studied, we witnessed a similar increase. Interestingly, urban women (57.4%) and educated women (56.8%) were much more likely to believe this than others, and young people just as likely as older people over to think this, with 44.2% of people under 25, thinking that condoms do promote promiscuity. Sex education was thought to be equally damaging, with more people agreeing that it promotes sexual activity and promiscuity in 2009 than in 2003 (19.3% vs. 30.2%, AOR 1.8; 95% CI 1.4-2.3, p<0.001). Women, especially urban women, were the most likely to think this and this feeling had grown over time (16.1% vs.33.8%; AOR 2.6; 95% CI 1.8-3.8, p=<0.001). Young people were just as likely as older people to agree with the statement and to have become more conservative over time. Agreement with this statement grew in all education groups.

## Discussion

Like the rest of India, Karnataka is changing rapidly due to a fast-growing economy and recent new investments in education and technology. The HIV epidemic is just one of the new social stressors and what we have observed in this study has to be seen in that light. The HIV epidemic remains a concentrated one with the burden of infection still in high risk groups and with little expansion into the general population [32]. News of the epidemic has spread into the community only recently, partly because the nature of the epidemic means that intervention efforts have mainly focused on high risk groups rather than on the general population. The HIV prevention program in Bagalkot has included some general population activities (such as sexuality and gender training of a few community members) and the creation of link workers whose job it is to educate the community and to be involved in village health committee activities. An earlier evaluation of the sexuality and gender training [33] showed that there had been little diffusion of information to the community at large, partly because the training did not include enough agency for participants to take the training further. Our study shows that by 2009, awareness of HIV in Bagalkot was almost universal. However, awareness of condoms and other prevention modalities was still not very high in 2009, even among the educated. In fact as HIV prevention strategies, the most educated people were more likely to mention the not very sensitive issue of injection avoidance, than limiting of the number of sexual partners, though this might merely reflect that participants do not want to reveal personal knowledge of such sensitive issues. In addition, attitudes to safe sexual practices such as masturbation were more negative in 2009, even among the well educated and young people, suggesting that sexually conservative mores had increased in this community.

Fear and stigma, however, appeared to have reduced significantly between 2003 and 2009. Fewer people in 2009 were superstitious about the role of God in punishing people with HIV, and fewer felt that HIV-infected people should be ostracised or stigmatized, showing that the community feels less fearful of the new epidemic than it may have done initially, and pointing to the success of the intervention programme. However, as in other studies [10,12,13,16,22-27] we observed that people increasingly blamed victims for the HIV epidemic (for example sex workers). Women and the more educated women were the most likely to feel this way, and young people appeared to be no more enlightened than older people. It is not clear, however, whether this “blaming” is just associated with increased awareness of vulnerable groups in their communities rather than to any real finger pointing, though the reducing levels of stigma might point to the former.

Studies have shown that when faced with a social stressor such as an HIV epidemic, many communities react conservatively and develop what has been described as moral and sexual panic, whipped up by the media, politicians and religious leaders [13-16,19,20]. In India as in other countries, there have been demonstrations instigated by politicians reacting to events such as films about lesbianism or that involve kissing, or practices (such as homosexuality, the sex trade, dressing inappropriately) that are deemed as culturally inappropriate, or threats to Indian mores and identity [20,34]. Often as a reaction to HIV authorities have developed education campaigns that promote unrealistic prevention methods, such as abstinence [16,20]. These are common reactions in India too, where the majority of people support “moral” education in schools (generally meaning abstinence promotion), but not “sex” education [4]. Evaluations of such programmes in many countries, however, have demonstrated that abstinence-only programmes have not been effective [35,36]; in addition, sex education that includes discussions of HIV and condoms does *not* increase promiscuity among those who are sexually naive but does have a positive effect on safe sex among those who are already sexually active [36-39]. Similarly, although the common prevailing wisdom in many societies is that condom availability promotes promiscuity [40], there is no evidence for this [37,41]. In a study by Das and colleagues, the authors assert that HIV has contributed to the inertia of sexual conservatism, because of the presumed negative effects of sexual liberalism [28]. Indeed in our study looking at attitudes over time, we found that there appeared to be increasingly less openness around discussing sex, AIDS and condoms; access to sex education and condoms were both increasingly thought to promote promiscuity. However, this conservative reaction might be the consequence of seeing widely promoted and sexually explicit condom packaging. In the study by Das et al., building on the work of others such as Kamwendo [42], the authors contend that HIV/AIDS as a social episode in a community influences some groups to become more sexually conservative than others. For example, more educated people are able to understand the negative meanings of sexual liberalism and react accordingly; young people are thought to react negatively because they are more vulnerable to HIV/AIDS and therefore might instinctively feel the need to protect themselves by holding conservative views about sexuality; and women might start to realize that sexual liberalism may affect them greatly [28]. In our study, we found indeed that young people, especially young women were the most resistant to change in terms of sexual conservatism around sex education and condom promotion. Level of education did not seem to influence responses to these questions, with just as much negativity in the most educated groups as in the least educated groups.

In summary, despite increased knowledge and positive changes around HIV-related stigma, resistance to change was apparent, with increased unwillingness to embrace openness and discuss sexuality. Young and educated respondents appeared to react as conservatively as others, reflecting a cultural inertia that mirrors studies of other pressures on traditional societal values. This is not to say that HIV intervention programming has been a failure, or that communities are in a heightened state of moral panic but that sexual conservatism is a natural reaction in communities that are pressured by a new social stressor. Progressive social change takes time, and without specific efforts to educate people so that they do not misunderstand the causes of the epidemic, conservative social reactions may occur. More effort is therefore required to educate young people in particular about healthy sexuality, openness and safe sex.

## Competing interests

The authors declare that they have no competing interests.

## Authors’ contributions

JB conceived of the study, supervised the analysis and wrote the manuscript; SR conducted the data analysis and commented on the manuscript; MA, SM, PB, RW and BMR provided support for data analysis and provided constructive feedback on the manuscript.

## Supplementary Material

Additional file 1 Measurements of knowledge and attitudinal change Click here for file

Additional file 2 Knowledge of HIV, condoms and sexual practices, by sex and location of residence, 2003 and 2009 Click here for file

Additional file 3 Knowledge of HIV, condoms and sexual practices, by age and education, 2003 and 2009 Click here for file

Additional file 4 Attitudes around HIV, by sex and location of residence, 2003 and 2009 Click here for file

Additional file 5 Attitudes around HIV, by age and education, 2003 and 2009 Click here for file

Additional file 6 Attitudes around sexuality, and around openness to discussing sexuality and HIV, by sex and location of residence, 2003 and 2009 Click here for file

Additional file 7 Attitudes around sexuality, and around openness to discussing sexuality and HIV, by age and education, 2003 and 2009 Click here for file
