# Supplementary material for: Changes in HIV knowledge, and socio-cultural and sexual attitudes in South India from 2003-2009
Source: BMC Public Health. 2011 Dec 29;11(Suppl 6):S12. doi: 10.1186/1471-2458-11-S6-S12 (PMC3287550; doi:10.1186/1471-2458-11-S6-S12)
Supplement: Additional file 1 — Measurements of knowledge and attitudinal change [file 1471-2458-11-S6-S12-S1.docx]

**Table 1: Measurements of knowledge and attitudinal change**

| **Hypotheses** | **Measurement** |
| --- | --- |
| **HIV/AIDS KNOWLEDGE INCREASES** |  |
| Knowledge of HIV and condoms increases | % who know about HIV increases |
|  | % who know about condoms increases |
| Knowledge about high risk HIV prevention increases | % who mention that condoms prevent transmission increases |
|  | % who mention that limiting to one partner prevents transmission increases |
| **KNOWLEDGE OF OTHER ASPECTS OF SEXUALITY INCREASES** | % who mention that masturbation is harmful to health falls |
| **BUT, BLAME AND PANIC ENSUES** |  |
| Issues around morality/religion increase | % who agree with the statement that AIDS is a punishment from God for sins committed increases |
| Fear of contagion and identification of “folk devils” increases, leading to stigmatization | % who agree with the statement that people with HIV should be thrown out of the community to stop the disease from spreading increases |
|  | % who agree that sex workers should be compulsorily tested for HIV increases |
|  | % who agree that a person who has sex outside marriage deserves to get AIDS increases |
|  | % who agree that children with HIV should have separate schools increases |
|  | % who agree that one should not take a bride from households with HIV infected persons increases |
| **CULTURAL INERTIA AND MORAL CODES INTENSIFY** | % who agree that a woman should be a virgin when she marries increases |
|  | % who agree that it is immoral for a woman to seek pleasure in sex increases |
|  | % who agree that it is wrong to talk about sex increases |
|  | % who agree that it is wrong to talk about AIDS in a respectable family increases |
|  | % who agree that it is not proper for a respectable person to talk about condoms increases |
|  | % who agree that easy access to condoms promotes promiscuity increases |
|  | % who agree that sex education increases sexual activity and promiscuity increases |
