# Supplementary material for: Changes in HIV knowledge, and socio-cultural and sexual attitudes in South India from 2003-2009
Source: BMC Public Health. 2011 Dec 29;11(Suppl 6):S12. doi: 10.1186/1471-2458-11-S6-S12 (PMC3287550; doi:10.1186/1471-2458-11-S6-S12)
Supplement: Additional file 2 — Knowledge of HIV, condoms and sexual practices, by sex and location of residence, 2003 and 2009 [file 1471-2458-11-S6-S12-S2.docx]

**Table 2: Knowledge of HIV, condoms and sexual practices, by sex and location of residence, 2003 and 2009**

| **% Respondents who**: | **RURAL (%)** | | | **URBAN (%)** | | | **TOTAL (%)** | | |
| --- | --- | --- | --- | --- | --- | --- | --- | --- | --- |
|  | **Male** | **Female** | **Total** | **Male** | **Female** | **Total** | **Male** | **Female** | **Total** |
| Have heard of HIV/AIDS  2003  2009  AOR (95% CI)  *P value* | 77.1  87.3  1.8(0.7-4.2)  0.16 | 68.5  81.4  1.9(0.67-5.5)  0.19 | 72.7  84.2  1.9(0.8-4.2)  0.12 | 91.2  93.9  1.4(0.7-2.6)  0.32 | 79.2  93.3  3.5(1.6-7.2)  0.002 | 84.9  93.6  2.4(1.3-4.7)  0.01 | 81.9  89.9  1.7(0.9-3.1)  0.09 | 72.2  86.0  2.2(1.1-4.6)  0.03 | 76.9  87.8  2.0 (1.1-3.5)  0.02 |
| Have heard of or seen condom  2003  2009  AOR (95% CI)  *P value* | 47.3  80.8  5.4 (2.7-10.5)  <0.001 | 12.9  42.2  5.1 (3.8-6.7)  <0.001 | 29.7  60.3  5.1(3.4-7.7)  <0.001 | 74.8  88.7  2.7(1.6-4.7)  0.001 | 32.4  59.0  3.2(2.4-4.3)  <0.001 | 52.7  73.4  3.0(2.3-3.9)  <0.001 | 56.5  84.0  4.4(2.8-6.8)  <0.001 | 19.6  48.6  4.3(3.6-5.3)  <0.001 | 37.4  65.4  4.3( 3.4-5.5)  <0.001 |
| Say spontaneously that condoms prevent HIV transmission  2003  2009  AOR (95% CI)  *P value* | 8.2  28.6  4.3(2.6-6.8)  <0.001 | 1.6  11.2  7.3(3.5-15.0)  <0.001 | 4.8  19.3  4.9(3.1-7.5)  <0.001 | 23.7  40.7  2.3(1.6-3.2)  <0.001 | 7.4  17.5  2.4(1.7-3.3)  <0.001 | 15.2  28.8  2.3(1.7-3.0)  <0.001 | 13.4  33.4  3.1(2.4-4.1)  <0.001 | 3.6  13.6  3.8(2.8-5.2)  <0.001 | 8.3  23.0  3.3(2.6-4.1)  <0.001 |
| Say spontaneously that limiting the no. partners prevents HIV transmission  2003  2009  AOR (95% CI)  *P value* | 41.0  23.0  0.4(0.1-1.0)  0.05 | 18.2  18.5  0.9(0.6-1.5)  0.72 | 29.3  20.6  0.6(0.3-1.0)  0.06 | 43.5  27.5  0.4(0.3-0.8)  0.09 | 28.5  27.5  0.8(0.6-1.1)  0.23 | 35.7  27.5  0.6(0.4-0.9)  0.08 | 41.9  24.8  0.4(0.2-0.7)  0.04 | 21.7  21.9  0.9(0.7-1.1)  0.33 | 31.5  23.3  0.6(0.4-0.8)  0.04 |
| Say spontaneously that avoiding injections prevents HIV transmission  2003  2009  AOR (95% CI)  *P value* | 18.7  19.9  0.9(0.4-1.7)  0.64 | 16.6  14.8  0.8(0.4-1.4)  0.33 | 17.6  17.2  0.8(0.5-1.3)  0.32 | 28.8  29.9  1.0(0.6-1.8)  0.95 | 24.6  22.5  0.7(0.5-1.0)  0.05 | 26.6  26.1  0.9(0.6-1.2)  0.41 | 22.1  23.9  0.9(0.6-1.4)  0.69 | 19.3  17.8  0.7(0.5-1.0)  0.08 | 20.7  20.7  0.8(0.6-1.1)  0.20 |
| Think masturbation harmful to health  2003  2009  AOR (95% CI)  *P value* | 40.3  49.3  1.5(0.7-3.3)  0.26 | 21.4  26.7  1.4(0.6-2.8)  0.37 | 30.6  37.3  1.4(0.7-2.9)  0.27 | 42.0  50.5  1.5(0.8-2.7)  0.24 | 19.7  28.6  1.6(1.1-2.4)  0.01 | 30.4  39.2  1.5(1.0-2.4)  0.06 | 40.9  49.8  1.5(0.9-2.4)  0.11 | 20.8  27.4  1.5(0.9-2.3)  0.08 | 30.5  38.1  1.5(1.0-2.3)  0.07 |
