# Supplementary material for: Changes in HIV knowledge, and socio-cultural and sexual attitudes in South India from 2003-2009
Source: BMC Public Health. 2011 Dec 29;11(Suppl 6):S12. doi: 10.1186/1471-2458-11-S6-S12 (PMC3287550; doi:10.1186/1471-2458-11-S6-S12)
Supplement: Additional file 3 — Knowledge of HIV, condoms and sexual practices, by age and education, 2003 and 2009 [file 1471-2458-11-S6-S12-S3.docx]

**Table 3: Knowledge of HIV, condoms and sexual practices, by age and education, 2003 and 2009**

| **% respondents who:** | **AGE (%)** | | | | | | | | | | | **YEARS OF EDUCATION (%)** | | | | | | | | | | | |  |
| --- | --- | --- | --- | --- | --- | --- | --- | --- | --- | --- | --- | --- | --- | --- | --- | --- | --- | --- | --- | --- | --- | --- | --- | --- |
|  | **Male** | | | **Female** | | | | **Total** | | | | **Male** | | | | **Female** | | | | **Total** | | | |  |
|  | **15-24** | **25-34** | **35-49** | | **15-24** | **25-34** | **35-49** | | **15-24** | **25-34** | **35-49** | | **<5** | **5-9** | **10+** | | **<5** | **5-9** | **10+** | | **<5** | **5-9** | **10+** | |
| Have heard of HIV/AIDS  2003  2009  AOR (95% CI)  *P value* | 81.2  90.5  1.6(0.9-3.0)  0.09 | 86.7  92.0  1.6(0.8-2.9)  0.17 | 77.7  87.2  1.8(0.8-3.8)  0.11 | | 73.3  86.8  1.9(0.9-4.1)  0.09 | 72.7  87.2  2.6(1.3-4.9)  0.06 | 70.4  84.0  2.3(0.9-5.8)  0.06 | | 77.2  88.6  1.8(1.0-3.2)  0.04 | 79.4  89.5  2.1(1.2-3.6)  0.007 | 73.9  85.4  2.1(0.9-4.4)  0.05 | | 67.6  76.8  1.6(0.7-3.4)  0.23 | 85.2  90.4  1.6(0.9-2.9)  0.10 | 95.5  98.1  2.6(1.5-4.4)  0.001 | | 63.1  77.8  2.3(1.0-5.1)  0.05 | 79.3  92.0  3.1(1.6-6.2)  0.02 | 94.3  95.8  1.3(0.5-3.4)  0.62 | | 64.8  77.4  2.0(1.0-3.9)  0.05 | 82.4  91.2  2.3(1.5-3.6)  0.001 | 95.1  97.2  1.8(1.0-3.4)  0.05 | |
| Have heard of, or seen condom  2003  2009  AOR (95% CI)  *P value* | 52.8  86.1  5.6(3.6-8.8)  <0.001 | 65.9  88.7  4.5(2.0-9.7)  <0.001 | 51.4  76.9  3.4(2.0-5.6)  <0.001 | | 20.3  49.6  3.6(2.7-4.9)  <0.001 | 23.6  56.2  5.0(3.8-6.7)  <0.001 | 14.6  40.9  4.8(3.3-7.1)  <0.001 | | 36.4  67.7  4.4(3.3-5.7)  <0.001 | 43.9  71.5  4.8(3.2-7.2)  <0.001 | 32.2  57.2  3.9(3.1-5.0)  <0.001 | | 30.9  66.6  4.9(2.7-8.9)  <0.001 | 59.5  83.7  3.7(2.4-5.6)  <0.001 | 82.8  95.2  4.7(2.7-8.4)  <0.001 | | 6.6  33.1  7.4(5.5-9.8)  <0.001 | 26.6  50.0  2.9(2.1-4.2)  <0.001 | 54.7  75.8  3.0(2.2-4.1)  <0.001 | | 15.9  44.9  5.9(4.2-8.2)  <0.001 | 43.9  66.4  3.2(2.3-4.4)  <0.001 | 72.8  87.6  3.6(2.6-4.8)  <0.001 | |
| Say spontaneously that condoms prevent HIV transmission  2003  2009  AOR (95% CI)  *P value* | 15.7  33.0  2.3(1.5-3.4)  <0.001 | 15.2  38.2  3.7(2.5-5.3)  <0.001 | 8.8  29.2  4.0(2.8-5.6)  <0.001 | | 3.3  13.9  3.9(2.5-5.9)  <0.001 | 2.1  11.2  3.1(2.0-4.7)  <0.001 | 9.4  23.4  5.5(3.0-10.0  <0.001 | | 9.4  23.4  2.6(1.9-3.7)  <0.001 | 10.0  26.4  3.5(2.5-4.7)  <0.001 | 5.3  19.4  4.3(3.1-5.9)  <0.001 | | 1.6  14.1  10.2(6.5-16.2)  <0.001 | 8.6  27.8  3.9(2.3-6.7)  <0.001 | 29.5  48.6  2.3(1.6-3.3)  <0.001 | | 0.4  7.6  21.3(7.1-63.9)  0<0.001 | 1.4  13.1  10.6(4.6-24.2)  <0.001 | 16.1  25.0  1.8(1.3-2.4)  <0.001 | | 0.8  9.9  13.7(9.2-20.5)  <0.001 | 5.2  20.2  4.9(3.1-7.7)  <0.001 | 24.8  39.3  2.2(1.6-2.9)  <0.001 | |
| Say spontaneously that limiting the no. partners prevents HIV transmission  2003  2009  AOR (95% CI)  *P value* | 32.5  16.8  0.4(0.2-0.6)  0.001 | 47.7  29.7  0.4(0.2-0.9)  0.02 | 48.1  29.5  0.4(0.2-0.8)  0.01 | | 24.9  19.3  0.6(0.4-0.8)  0.001 | 21.9  24.4  1.0(0.7-1.5)  0.92 | 18.1  22.4  1.2(0.8-1.7)  0.31 | | 28.6  18.0  0.5(0.3-0.7)  <0.001 | 33.9  26.9  0.7(0.4-1.0)  0.05 | 32.4  25.7  0.7(0.4-1.0)  0.06 | | 37.3  19.4  0.4(0.1-0.9)  0.04 | 43.2  22.9  0.3(0.2-0.7)  0.004 | 46.0  29.3  0.5(0.3-0.8)  0.002 | | 12.7  17.5  1.4(0.9-2.3)  0.13 | 26.9  22.6  0.8(0.6-1.0)  0.14 | 46.0  29.6  0.5(0.4-0.6)  <0.001 | | 22.1  18.1  0.8(0.5-1.2)  0.25 | 35.5  22.8  0.5(0.3-0.8)  0.008 | 46.0  29.3  0.5(0.4-0.6)  <0.001 | |
| Say spontaneously that avoiding injections prevents HIV transmission  2003  2009  AOR (95% CI)  *P value* | 2.3  23.6  0.8(0.5-1.4)  0.43 | 26.2  25.1  0.8(0.5-1.4)  0.50 | 17.7  22.9  1.1(0.7-1.8)  0.65 | | 21.7  17.6  0.6(0.4-0.9)  0.02 | 18.0  20.9  1.0(0.7-1.7)  0.86 | 18.1  15.1  0.6(0.4-1.0)  0.06 | | 22.0  20.6  0.7(0.5-1.0)  0.04 | 21.9  22.9  0.9(0.7-1.3)  0.73 | 17.9  18.7  0.9(0.6-1.2)  0.38 | | 8.6  11.6  1.4(0.7-2.6)  0.32 | 21.5  20.7  0.9(0.5-1.4)  0.56 | 37.7  33.4  0.8(0.5-1.3)  0.34 | | 8.8  11.2  1.3(0.7-2.5)  0.42 | 25.7  15.9  0.5(0.4-0.8)  0.003 | 47.0  31.4  0.5(0.3-0.8)  0.003 | | 8.7  11.3  1.4(0.9-2.0)  0.12 | 23.5  18.2  0.7(0.5-0.9)  0.02 | 41.0  32.6  0.7(0.5-1.0)  0.05 | |
| Think masturbation harmful to health  2003  2009  AOR (95% CI)  *P value* | 36.5  47.9  1.6(1.0-2.6)  0.05 | 45.2  56.2  1.6(1.0-2.7)  0.07 | 41.9  45.8  1.3(0.7-2.3)  0.46 | | 15.9  26.5  2.0(1.2-3.5)  0.01 | 23.4  28.8  1.4(0.8-2.2)  0.18 | 23.8  27.1  1.2(0.8-1.9)  0.41 | | 26.1  37.1  1.8(1.1-2.7)  0.01 | 33.8  41.8  1.5(1.0-2.3)  0.07 | 32.4  35.6  1.2(0.8-1.9)  0.38 | | 37.1  47.7  1.6(0,7-4.1)  0.28 | 43.7  54.4  1.6(1.0-2.6)  0.7 | 43.3  48.8  1.3(0.9-1.9)  0.10 | | 21.3  26.3  1.4(0.7-2.7)  0.36 | 20.8  28.1  1.6(1.1-2.2)  0.01 | 19.4  28.8  1.7(1.2-2.4)  0.005 | | 27.3  33.8  1.5(0.7-3.1)  0.29 | 32.8  40.9  1.6(1.1-2.3)  0.01 | 34.8  40.9  1.4(1.1-1.9)  0.01 | |
