# Supplementary material for: Changes in HIV knowledge, and socio-cultural and sexual attitudes in South India from 2003-2009
Source: BMC Public Health. 2011 Dec 29;11(Suppl 6):S12. doi: 10.1186/1471-2458-11-S6-S12 (PMC3287550; doi:10.1186/1471-2458-11-S6-S12)
Supplement: Additional file 4 — Attitudes around HIV, by sex and location of residence, 2003 and 2009 [file 1471-2458-11-S6-S12-S4.docx]

**Table 4: Attitudes around HIV, by sex and location of residence, 2003 and 2009**

| **% respondents who agree that** | **RURAL (%)** | | | **URBAN (%)** | | | | | **TOTAL (%)** | | |
| --- | --- | --- | --- | --- | --- | --- | --- | --- | --- | --- | --- |
|  | **Male** | **Female** | **Total** | | **Male** | **Female** | **Total** | **Male** | | **Female** | **Total** |
| AIDS is a punishment from God for sins committed  2003  2009  AOR (95% CI)  *P value* | 42.4  30.8  0.6(0.4-0.9)  0.02 | 55.4  35.2  0.5(0.3-0.7)  0.004 | 48.7  33.1  0.5(0.4-0.7)  <0.001 | | 27.5  21.5  0.7(0.5-1.0)  0.08 | 42.9  24.3  0.4(0.3-0.7)  0.001 | 35.0  22.9  0.6(0.4-0.7)  <0.001 | 36.8  26.9  0.6(0.5-0.9)  0.003 | | 50.7  30.7  0.5(0.3-0.6)  <0.001 | 43.5  28.9  0.5(0.5-0.6)  <0.001 |
| A person who has sex outside the marriage deserves to get AIDS  2003  2009  AOR (95% CI)  *P value* | 61.0  59.7  0.9(0.6-1.4)  0.66 | 58.9  63.5  1.1(0.7-1.9)  0.55 | 60.0  61.6  1.0(0.7-1.4)  0.91 | | 69.2  56.6  0.6(0.4-0.8)  0.006 | 59.0  62.0  1.1(0.7-1.6)  0.65 | 64.3  59.4  0.8(0.6-1.1)  0.18 | 64.1  58.4  0.8(0.6-1.0)  0.07 | | 58.9  62.9  1.1(0.8-1.5)  0.43 | 61.6  60.7  0.9(0.7-1.1)  0.49 |
| Sex workers should be compulsorily tested for HIV  2003  2009  AOR (95% CI)  *P value* | 85.0  79.1  0.7(0.3-1.3)  0.18 | 72.4  85.1  2.0(1.3-3.0)  0.004 | 78.9  82.2  1.2(0.8-1.7)  0.38 | | 90.9  83.4  0.5(0.3-0.8)  0.004 | 82.8  88.3  1.5(1.1-2.1)  0.01 | 87.0  85.9  0.9(0.7-1.2)  0.41 | 87.2  80.9  0.6(0.4-0.9)  0.02 | | 76.3  86.4  1.8(1.4-2.4)  <0.001 | 81.9  83.7  1.0(0.8-1.4)  0.60 |
| People with HIV should be thrown out of the community to stop the disease from spreading  2003  2009  AOR (95% CI)  *P value* | 41.2  28.9  0.5(0.3-0.8)  0.01 | 51.3  34.0  0.5(03-07)  0.003 | 46.1  31.5  0.5(0.4-0.7)  0.001 | | 27.7  19.5  0.6(0.5-0.9)  0.007 | 41.0  25.8  0.5(0.4-0.7)  <0.001 | 34.2  22.7  0.6(0.5-0.6)  <0.001 | 36.1  25.0  0.6(0.4-0.7)  <0.001 | | 47.4  30.6  0.5(0.4-0.6)  <0.001 | 41.6  27.9  0.5(0.5-0.6)  <0.001 |
| One should not take a bride from a home with HIV-infected persons  2003  2009  AOR (95% CI)  *P value* | 42.5  37.1  0.8(0.5-1.3)  0.39 | 64.2  32.8  0.3(0.2-0.3)  <0.001 | 53.0  34.9  0.5(0.4-0.6)  <0.001 | | 34.2  25.3  0.7(0.5-0.8)  <0.001 | 54.2  30.9  2.4(0.3-0.5)  <0.001 | 43.9  28.2  0.5(0.4-0.6)  <0.001 | 39.4  32.2  0.8(0.6-1.0)  0.06 | | 60.4  32.0  0.3(0.3-0.4)  <0.001 | 49.5  32.1  0.5(0.4-0.6)  <0.001 |
| HIV positive children should have separate schools  2003  2009  AOR (95% CI)  *P value* | 41.8  30.4  0.6(0.4-0.8)  0.008 | 51.0  30.0  0.4(0.3-0.6)  <0.001 | 46.2  30.2  0.5(0.4-0.6)  <0.001 | | 30.2  24.5  0.8(0.5-1.1)  0.13 | 39.0  25.8  0.6(0.4-0.8)  0.001 | 34.5  25.2  0.6(0.6-0.7)  <0.001 | 37.4  27.9  0.6(0.5-0.8)  0.001 | | 46.5  28.2  0.5(0.4-0.6)  <0.001 | 41.8  28.2  0.5(0.5-0.6)  <0.001 |
