# Supplementary material for: Changes in HIV knowledge, and socio-cultural and sexual attitudes in South India from 2003-2009
Source: BMC Public Health. 2011 Dec 29;11(Suppl 6):S12. doi: 10.1186/1471-2458-11-S6-S12 (PMC3287550; doi:10.1186/1471-2458-11-S6-S12)
Supplement: Additional file 5 — Attitudes around HIV, by age and education, 2003 and 2009 [file 1471-2458-11-S6-S12-S5.docx]

**Table 5: Attitudes around HIV, by age and education, 2003 and 2009**

| **% respondents who agree that** | **AGE (%)** | | | | | | | | | | | | **YEARS OF EDUCATION (%)** | | | | | | | | | | |
| --- | --- | --- | --- | --- | --- | --- | --- | --- | --- | --- | --- | --- | --- | --- | --- | --- | --- | --- | --- | --- | --- | --- | --- |
|  | **Male** | | | | **Female** | | | | **Total** | | | | **Male** | | | | **Female** | | | | **Total** | | |
|  | **15-24** | **25-34** | **35-49** | **15-24** | | **25-34** | **35-49** | **15-24** | | **25-34** | **35-49** | **<5** | | **5-9** | **10+** | **<5** | | **5-9** | **10+** | **<5** | | **5-9** | **10+** |
| AIDS is a punishment from God for sins committed  2003  2009  AOR (95% CI)  *P value* | 37.2  26.1  0.6(0.4-0.8)  0.002 | 36.4  25.4  0.6(0.4-0.9)  0.01 | 36.7  29.5  0.8(0.5-1.2)  0.24 | 43.9  28.0  0.5(0.4-0.8)  0.002 | | 52.2  30.1  0.4(0.3-0.6)  <0.001 | 57.2  34.0  0.4(03-0.5)  <0.001 | 40.4  27.1  0.6(0.4-0.7)  <0.001 | | 43.9  27.8  0.5(0.4-0.6)  <0.001 | 46.9  31.9  0.6(0.4-0.7)  <0.001 | 47.0  31.7  0.5(0.4-0.7)  <0.001 | | 34.0  30.4  0.8(0.5-1.5)  0.49 | 30.3  22.9  0.7(0.5-0.9)  0.01 | 61.7  39.0  0.4(0.3-0.6)  <0.001 | | 48.8  28.9  0.4(0.3-0.6)  <0.001 | 28.3  19.8  0.6(0.4-0.9)  0.01 | 55.8  36.4  0.4(04-0.5)  <0.001 | | 40.8  29.6  0.6(0.4-0.8)  0.001 | 29.6  21.7  0.6(0.5-0.8)  0.001 |
| A person who has sex outside the marriage deserves to get AIDS  2003  2009  AOR (95% CI)  *P value* | 62.9  56.3  0.7(0.5-1.2)  0.19 | 62.6  61.0  0.9(0.7-1.2)  0.60 | 67.3  58.4  0.6(0.5-0.9)  0.006 | 59.3  62.3  1.1(0.8-1.7)  0.54 | | 61.9  63.7  1.0(0.7-1.5)  0.91 | 55.5  62.7  1.2(0.9-1.8)  0.24 | 61.1  59.3  0.9(0.7-1.2)  0.49 | | 62.2  62.4  097(0.8-1.2)  0.83 | 61.4  60.7  0.9(0.7-1.2)  0.44 | 61.2  59.9  0.9(0.6-1.4)  0.67 | | 65.6  55.5  0.6(0.4-0.8)  0.005 | 65.5  59.1  0.8(0.5-1.1)  0.16 | 55.4  63.8  1.3(0.9-1.8)  0.20 | | 63.5  57.4  0.7(0.5-1.2)  0.21 | 62.6  66.0  1.2(0.8-1.8)  0.35 | 57.7  62.4  1.1(0,8-1.5)  0.44 | | 64.6  56.5  0.7(0.5-0.9)  0.008 | 64.5  61.8  0.9(0.7-1.2)  0.55 |
| Sex workers should be compulsorily tested for HIV  2003  2009  AOR (95% CI)  *P value* | 83.6  79.4  0.7(0.4-1.2)  0.18 | 88.4  84.1  0.7(0.4-1.2)  0.18 | 90.5  79.3  0.4(0.2-0.8)  0.007 | 76.1  86.8  2.0(1.4-2.7)  <0.001 | | 79.0  85.6  1.4(0.9-2.1)  0.09 | 73.9  86.8  2.1(1.3-3.3)  0.002 | 80.0  83.1  1.2(0.9-1.5)  0.32 | | 83.9  84.9  1.0(0.7-1.5)  0.96 | 82.3  83.3  0.9(0.6-1.5)  0.95 | 80.8  77.1  0.8(0.5-1.5)  0.48 | | 88.8  77.7  0.4(0.3-0.7)  0.001 | 91.3  84.3  0.5(0.3-0.9)  0.02 | 70.2  81.8  1.8(1.3-2.7)  0.002 | | 79.3  88.3  1.9(1.1-3.3)  0.02 | 87.2  91.8  1.7(1.1-2.4)  0.01 | 74.5  80.2  1.4(1.0-1.8)  0.03 | | 84.4  83.2  2.9(0.6-1.3)  0.56 | 89.9  87.2  0.8(0.6-1.1)  0.13 |
| People with HIV should be thrown out of the community to stop the disease from spreading  2003  2009  AOR (95% CI)  *P value* | 38.3  27.0  0.6(0.4-0.9)  0.01 | 36.6  21.1  0.4(0.3-0.7)  <0.001 | 32.9  26.6  0.7(0.5-1.0)  0.04 | 44.2  26.0  0.5(0.3-0.7)  0.001 | | 47.5  31.1  0.5(0.4-0.7)  0.001 | 51.2  34.9  0.5(0.4-0.6)  <0.001 | 41.4  26.5  0.5(0.4-0.7)  <0.001 | | 41.8  26.2  0.5(0.3-0.6)  <0.001 | 42.0  31.0  0.6(0.5-0.7)  <0.001 | 41.5  36.9  0.7(0.5-1.0)  0.07 | | 41.6  26.1  0.4(0.3-0.7)  0.003 | 28.9  18.5  0.5(0.4-0.7)  <0.001 | 54.6  39.9  0.5(0.4-0.7)  <0.001 | | 46.9  28.9  0.5(0.3-0.7)  0.002 | 32.1  18.0  0.5(0.3-0.7)  <0.001 | 49.4  38.9  0.6(0.5-0.8)  <0.001 | | 44.0  27.5  0.5(0.3-0.7)  <0.001 | 30.1  18.4  0.5(04-0.6)  <0.001 |
| One should not take a bride from a home with HIV-infected persons  2003  2009  AOR (95% CI)  *P value* | 40.1  32.6  0.8(0.6-1.1)  0.11 | 38.2  30.9  0.7(0.5-1.1)  0.14 | 39.7  33.0  0.8(0.6-1.0)  0.09 | 54.9  28.8  0.4(0.3-0.5)  <0.001 | | 60.5  32.4  0.3(0.3-0.4)  <0.001 | 66.9  35.1  0.3(0.2-0.3)  <0.001 | 47.2  30.7  0.5(0.4-0.7)  <0.001 | | 48.9  31.7  0.5(0.4-0.6)  <0.001 | 53.2  34.1  0.4(0.4-0.5)  <0.001 | 44.1  42.3  0.9(0.7-1.3)  0.62 | | 42.7  33.1  0.7(0.5-0.9)  0.02 | 33.8  26.7  0.7(0.5-1.0)  0.06 | 67.6  41.0  0.3(0.3-0.4)  <0.001 | | 58.5  28.0  0.3(0.2-0.4)  <0.001 | 46.4  22.0  0.3(0.2-0.4)  <0.001 | 58.2  41.4  0.5(0.4-0.6)  <0.001 | | 49.9  30.5  0.4(0.3-0.6)  <0.001 | 38.2  24.9  0.5(0.4-0.7)  <0.001 |
| HIV positive children should have separate schools  2003  2009  AOR (95% CI)  *P value* | 38.6  27.5  0.6(.05-0.8)  0.001 | 36.2  25.4  0.6(0.4-0.9)  0.007 | 37.5  31.0  0.7(0.5-1.0)  0.08 | 44.7  22.5  0.4(0.3-0.5)  <0.001 | | 47.4  30.2  0.5(0.4-0.7)  <0.001 | 47.7  32.3  0.5(0.4-0.7)  <0.001 | 41.5  25.0  0.5(0.4-0.6)  <0.001 | | 41.5  27.9  0.5(0.4-0.7)  <0.001 | 42.6  31.7  0.6(0.5-0.7)  <0.001 | 41.9  38.2  0.8(0.5-1.1)  0.18 | | 42.1  29.7  0.6(0.4-0.8)  0.007 | 31.3  21.9  0.6(0.4-0.8)  <0.001 | 53.5  35.1  0.5(0.4-0.6)  <0.001 | | 47.1  28.7  0.4(0.3-0.7)  <0.001 | 30.6  17.7  0.5(0.4-0.7)  <0.001 | 48.9  36.2  0.5(0.5-0.7)  <0.001 | | 44.4  29.2  0.5(0.4-0.7)  <0.001 | 31.0  20.3  0.6(0.4-0.7)  <0.001 |
