# Supplementary material for: Changes in HIV knowledge, and socio-cultural and sexual attitudes in South India from 2003-2009
Source: BMC Public Health. 2011 Dec 29;11(Suppl 6):S12. doi: 10.1186/1471-2458-11-S6-S12 (PMC3287550; doi:10.1186/1471-2458-11-S6-S12)
Supplement: Additional file 6 — Attitudes around sexuality, and around openness to discussing sexuality and HIV, by sex and location of residence, 2003 and 2009 [file 1471-2458-11-S6-S12-S6.docx]

**Table 6: Attitudes around sexuality, and around openness to discussing sexuality and HIV, by sex and location of residence, 2003 and 2009**

| % respondents who agree that | **RURAL (%)** | | | **URBAN (%)** | | | **TOTAL (%)** | | |
| --- | --- | --- | --- | --- | --- | --- | --- | --- | --- |
|  | **Male** | **Female** | **Total** | **Male** | **Female** | **Total** | **Male** | **Female** | **Total** |
| Women should be virgins at marriage  2003  2009  AOR (95% CI)  *P value* | 87.3  80.7  0.6(0.4-0.8)  0.004 | 92.2  93.2  1.2(0.6-2.2)  0.62 | 89.8  87.3  0.7(0.5-1.0)  0.08 | 89.8  84.6  0.6(0.4-1.0)  0.05 | 91.5  90.4  0.9(0.5-1.4)  0.49 | 90.7  87.6  0.7(0.5-1.1)  0.09 | 88.2  82.2  0.6(0.5-0.8)  <0.001 | 91.9  92.1  1.0(0.7-1.5)  0.85 | 90.1  87.4  0.7(0.6-0.9)  0.01 |
| It is immoral for women to seek pleasure in sex  2003  2009  AOR (95% CI)  *P value* | 12.8  17.4  1.5(0.7-2.9)  0.24 | 34.1  19.1  0.5(0.3-0.9)  0.02 | 23.7  18.3  0.7(0.4-1.2)  0.21 | 8.0  10.3  1.3(0.8-2.1)  0.26 | 31.8  11.0  0.3(0.2-0.4)  <0.001 | 20.4  10.7  0.5(0.3-0.6)  <0.001 | 11.2  14.6  1.4(0.9-2.2)  0.14 | 33.3  6.0  0.4(0.3-0.6)  <0.001 | 22.6  15.3  0.6(0.5-0.9)  0.01 |
| It is wrong to talk about sex  2003  2009  AOR (95% CI)  *P value* | 30.5  29.2  0.9(0.4-2.2)  0.90 | 22.5  34.2  1.9(1.2-3.0)  0.008 | 26.4  31.8  1.4(0.9-2.0)  0.09 | 18.7  20.2  1.1(0.7-1.8)  0.64 | 20.7  29.5  1.7(1.2-2.4)  0.008 | 19.8  25.0  1.4(0.9-2.0)  0.07 | 26.6  25.6  1.0(0.6-1.7)  0.98 | 21.9  32.4  1.9(1.4-2.5)  <0.001 | 24.2  29.2  1.4(1.1-1.9)  0.02 |
| It is wrong to talk about AIDS in a respectable family  2003  2009  AOR (95% CI)  *P value* | 18.3  24.9  1.4(0.7-2.9)  0.28 | 29.0  29.8  1.2(0.6-2.7)  0.52 | 23.5  27.5  1.3(0.9-1.9)  0.16 | 16.9  19.7  1.2(08-2.0)  0.37 | 18.7  24.3  1.5(1.1-2.0)  0.01 | 17.8  22.1  1.4(1.1-1.8)  0.02 | 17.8  22.7  1.4(0.9-2.1)  0.17 | 25.1  27.6  1.3(0.8-2.1)  0.23 | 21.4  25.2  1.3(1.0-1.7)  0.03 |
| It is not proper for a respectable person to talk about condoms  2003  2009  AOR (95% CI)  *P value* | 12.6  28.5  2.6(1.0-6.8)  0.04 | 22.4  31.6  1.6(0.8-3.2)  0.13 | 14.7  29.7  2.4(1.2-4.7)  0.02 | 14.3  21.8  1.6(1.1-2.4)  0.01 | 21.3  28.1  1.4(0.9-2.4)  0.14 | 16.6  24.4  1.6(1.1-2.1)  0.008 | 13.3  25.7  2.2(1.3-3.5)  0.004 | 21.8  30.0  1.5(1.0-2.3)  0.03 | 15.6  27.4  1.9(1.4-2.8)  0.001 |
| Easy access to condoms promotes promiscuity  2003  2009  AOR (95% CI)  *P value* | 30.2  41.2  1.5(0.8-2.8)  0.15 | 35.5  47.5  2.0(1.0-4.0)  0.05 | 31.3  43.5  1.6(0.9-2.7)  0.07 | 26.6  40.1  1.8(1.4-2.4)  <0.001 | 41.8  57.4  1.9(1.3-2.9)  0.002 | 31.5  47.3  1.9(1.5-2.3)  <0.001 | 28.6  40.7  1.6(1.2-2.3)  0.006 | 39.0  52.1  1.9(1.4-2.8)  <0.001 | 31.4  45.2  1.7(1.3-2.3)  <0.001 |
| Sex education increases sexual activity and promiscuity  2003  2009  AOR (95% CI)  *P value* | 20.3  28.2  1.5(0.8-2.7)  0.15 | 20.3  32.0  1.9(1.0-3.8)  0.006 | 20.3  30.2  1.7(1.2-2.4)  0.005 | 18.3  26.4  1.6(1.1-2.3)  0.02 | 16.1  33.8  2.6(1.8-3.8)  <0.001 | 17.1  30.2  2.1(1.5-2.9)  <0.001 | 19.7  27.4  1.5(1.0-2.2)  0.03 | 18.9  32.7  2.2(1.4-3.3)  0.001 | 19.3  30.2  1.8(1.4-2.3)  <0.001 |
