# Supplementary material for: Changes in HIV knowledge, and socio-cultural and sexual attitudes in South India from 2003-2009
Source: BMC Public Health. 2011 Dec 29;11(Suppl 6):S12. doi: 10.1186/1471-2458-11-S6-S12 (PMC3287550; doi:10.1186/1471-2458-11-S6-S12)
Supplement: Additional file 7 — Attitudes around sexuality, and around openness to discussing sexuality and HIV, by age and education, 2003 and 2009 [file 1471-2458-11-S6-S12-S7.docx]

**Table 7: Attitudes around sexuality, and around openness to discussing sexuality and HIV, by age and education, 2003 and 2009**

| **% respondents who agree that** | **AGE (%)** | | | | | | | | | | | **YEARS OF EDUCATION (%)** | | | | | | | | | | |
| --- | --- | --- | --- | --- | --- | --- | --- | --- | --- | --- | --- | --- | --- | --- | --- | --- | --- | --- | --- | --- | --- | --- |
|  | Male | | | Female | | | | | Total | | | Male | | | | Female | | | Total | | | |
|  | **15-24** | **25-34** | **35-49** | | **15-24** | **25-34** | **35-49** | **15-24** | | **25-34** | **35-49** | | **<5** | **5-9** | **10+** | **<5** | **5-9** | **10+** | | **<5** | **5-9** | **10+** |
| Women should be virgins at marriage  2003  2009  AOR (95% CI)  *P value* | 84.5  81.0  0.7(0.5-1.0)  0.05 | 90.3  85.6  0.6(0.3-1.1)  0.07 | 90.5  80.4  0.4(0.3-0.6)  <0.001 | | 89.0  92.4  1.6(0.8-2.9)  0.15 | 95.8  91.9  0.5(0.3-0.9)  0.02 | 91.4  92.0  1.0(0.6-1.6)  0.95 | 86.8  86.8  1.0(0.8-1.3)  0.90 | | 93.2  88.9  0.6(0.4-0.9)  0.02 | 91.0  86.7  0.6(0.4-0.9)  0.05 | | 86.7  80.2  0.6(0.4-0.8)  0.04 | 85.1  82.2  0.7(0.5-1.0)  0.08 | 91.7  83.5  0.5(0.3(0.7)  0.001 | 91.3  92.7  1.1(0.7-1.9)  0.59 | 92.0  90.5  0.8(0.4-1.6)  0.5 | 91.8  92.4  1.1(0.7-1.7)  0.79 | | 89.9  88.3  0.8(0.6-1.1)  0.14 | 88.4  86.5  0.8(0.5-1.2)  0.19 | 91.7  87.0  0.6(0.4-0.9)  0.008 |
| It is immoral for women to seek pleasure in sex  2003  2009  AOR (95% CI)  *P value* | 10.6  11.6  1.0(0.7-1.6)  0.90 | 13.2  16.7  1.4(0.9-2.4)  0.16 | 9.8  16.0  1.8(0.9-3.4)  0.07 | | 25.9  13.4  0.5(0.3-0.8)  0.005 | 40.0  18.1  0.3(0.2-0.6)  <0.001 | 34.9  16.8  0.4(0.2-0.6)  <0.001 | 18.4  12.5  0.7(0.5-0.9)  0.03 | | 27.2  17.5  0.6(0.4-0.9)  0.009 | 22.9  16.4  0.7(0.5-0.9)  0.02 | | 12.2  17.7  1.6(0.9-2.8)  0.13 | 7.9  13.8  1.8(1.0-3.1)  0.04 | 12.1  12.9  1.1(0.6-2.0)  0.70 | 37.5  19.8  0.4(0.3-0.6)  <0.001 | 31.1  13.6  0.4(0.2-0.6)  0.001 | 21.9  11.2  0.4(0.3-0.7)  0.003 | | 27.8  19.0  0.6(0.4-0.8)  0.001 | 18.9  13.7  0.6(0.4-1.0)  0.04 | 15.5  12.3  0.8(0.5-1.3)  .34 |
| It is wrong to talk about sex  2003  2009  AOR (95% CI)  *P value* | 26.5  26.7  1.1(0.6-1.9)  0.82 | 23.9  22.7  1.0(0.5-2.0)  0.99 | 29.3  27.1  0.9(0.5-1.6)  0.67 | | 21.4  31.1  1.8(1.2-2.7)  0.007 | 22.4  33.4  1.9(1.3-2.7)  0.002 | 22.0  32.8  1.9(1.4-2.5)  <0.001 | 24.0  28.9  1.4(0.9-2.1)  0.13 | | 23.1  28.3  1.4(1.1-1.8)  0.009 | 25.5  30.2  1.3(1.0-1.8)  0.07 | | 32.7  31.9  0.9(0.5-1.7)  0.86 | 24.1  26.5  1.1(0.6-2.2)  0.67 | 21.4  21.1  0.9(0.5-1.6)  0.85 | 22.7  37.9  2.1(1.5-3.1)  <0.001 | 20.5  32.5  1.9(1.3-3.0)  0.004 | 20.9  22.2  1.1(0.7-1.7)  0.62 | | 26.5  35.8  1.6(1.3-1.9)  <0.001 | 22.4  29.5  `1.5(0.9-2.3)  0.09 | 21.2  21.5  1.0(0.7-1.5)  0.95 |
| It is wrong to talk about AIDS in a respectable family  2003  2009  AOR (95% CI)  *P value* | 18.4  28.2  1.8(1.1-3.0)  0.02 | 17.6  17.1  0.9(0.5-1.7)  0.91 | 17.3  21.9  1.3(0.8-2.2)  0.25 | | 18.3  27.1  1.8(1.2-2.9)  0.009 | 29.6  28.2  1.1(0.6-2.1)  0.74 | 28.6  27.4  1.1(0.7-1.9)  0.66 | 18.7  24.3  1.8(1.3-2.6)  0.002 | | 23.3  22.8  1.0(0.7-1.4)  0.90 | 23.0  24.9  1.2(0.9-1.7)  0.26 | | 20.8  26.7  1.2(0.7-2.1)  0.4 | 17.8  25.3  1.6(0.8-3.3)  0.16 | 15.4  19.5  1.3(0.8-1.9)  0.24 | 31.2  30.7  1.2(0.7-1.9)  0.58 | 24.8  26.4  1.2(0.7-1.9)  0.57 | 12.2  23.8  2.3(1.5-3.4)  <0.001 | | 27.1  29.3  1.2(0.8-1.7)  0.40 | 21.0  25.9  1.3(0.9-1.9)  0.10 | 14.3  21.2  1.6(1.2-2.2)  0.003 |
| It is not proper for a respectable person to talk about condoms  2003  2009  AOR (95% CI)  *P value* | 13.5  26.2  2.2(1.2-4.2)  0.02 | 12.4  23.7  2.1(1.2-3.7)  0.01 | 14.4  27.3  2.2(1.2-4.0)  0.01 | | 24.6  28.6  1.3(0.7-2.5)  0.42 | 22.6  31.4  1.6(1.1-2.3)  0.02 | 16.0  30.0  2.2(1.2-4.2)  0.02 | 16.6  27.1  1.9(1.1-3.2)  0.02 | | 15.3  26.9  1.9(1.3-2.7)  0.001 | 14.8  28.3  2.2(1.4-3.5)  0.001 | | 12.8  30.8  2.7(1.4-5.0)  0.003 | 14.5  23.9  1.8(0.9-3.4)  0.08 | 13.1  24.2  2.1(1.2-3.50  0.009 | 15.9  33.8  2.7(1.6-4.7)  0.001 | 32.2  30.3  0.9(0.6-1.6)  0.85 | 18.8  26.7  1.7(0.9-3.0)  0.09 | | 13.6  32.2  2.9(1.9-4.5)  <0.001 | 19.6  26.4  1.4(0.9-2.2)  0.14 | 14.6  25.1  1.9(1.2-3.0)  0.005 |
| Easy access to condoms promotes promiscuity  2003  2009  AOR (95% CI)  *P value* | 31.8  39.6  1.3(0.8-2.1)  0.20 | 26.7  40.0  1.7(1.2-2.4)  0.002 | 27.0  43.0  1.9(1.3-2.9)  0.002 | | 37.9  52.2  1.8(1.0-3.3)  0.04 | 39.4  56.1  2.4(1.5-3.9)  0.001 | 40.4  46.9  1.8(1.2-2.7)  0.008 | 33.5  44.2  1.5(1.0-2.2)  0.04 | | 30.3  46.7  1.9(1.4-2.6)  <0.001 | 30.2  44.5  1.8(1.3-2.5)  <0.001 | | 28.5  38.6  1.6(0.8-3.0)  0.17 | 32.9  43.4  1.5(1.0-2.2)  0.04 | 26.7  40.4  1.8(1.2-2.5)  0.003 | 22.2  44.7  3.1(1.9-5.2)  <0.001 | 32.1  54.3  2.7(1.4-5.2)  0.005 | 49.2  56.8  1.5(0.9-2.3)  0.07 | | 26.9  41.6  1.9(1.1-3.0)  0.02 | 32.6  47.6  1.8(1.3-2.5)  0.002 | 32.7  46.0  1.7(1.2-2.3)  0.002 |
| Sex education increases sexual activity and promiscuity  2003  2009  AOR (95% CI)  *P value* | 16.6  26.8  1.8(1.1-2.9)  0.02 | 22.5  31.2  1.6(1.0-2.3)  0.04 | 20.5  24.6  1.2(0.8-1.8)  0.44 | | 15.2  30.1  2.6(1.5-4.5)  0.001 | 22.3  34.9  1.8(1.1-3.1)  0.02 | 19.5  33.4  2.1(1.4-3.3)  0.002 | 15.9  28.5  2.2(1.6-3.0)  <0.001 | | 22.4  33.1  1.7(1.3-2.2)  <0.001 | 20.0  29.3  1.6(1.2-2.2)  0.001 | | 17.7  25.3  1.6(0.9-2.8)  0.11 | 19.2  31.9  1.9(1.2-2.9)  0.007 | 22.1  26.5  1.3(0.9-1.9)  0.23 | 19.1  32.6  2.0(1.1-3.8)  0.02 | 18.3  33.1  2.4(1.4-3.9)  0.001 | 18.7  32.6  2.2(1.5-3.1)  <0.001 | | 18.6  30.0  1.9(1.3-2.6)  0.001 | 18.8  32.5  2.1(1.5-2.9)  <0.001 | 20.9  28.9  1.6(1.1-2.2)  0.008 |
